# Emphysematous Gastritis in the Setting of Stage Four Colon Cancer After Radiation Therapy and Ongoing Chemotherapy: A Case Report

**DOI:** 10.7759/cureus.91398

**Published:** 2025-09-01

**Authors:** Rollin William Johnson, Kofi Obeng, Hector DePaz, Mohammad Gilani, Kashif Saeed

**Affiliations:** 1 General Surgery, Wyckoff Heights Medical Center, New York, USA

**Keywords:** adenocarcinoma of colon, colon adenocarcinoma, conservative treatment, cytarabine, diagnostic laparoscopy, emphysematous gastritis, portal venous air

## Abstract

Emphysematous gastritis (EG) is a rare, life-threatening condition characterized by air in the stomach wall due to gas-producing bacteria, often accompanied by portal venous air. This case report describes a 50-year-old male with stage 4 colon cancer, undergoing cytarabine chemotherapy, who presented with sepsis, altered mental status, and melena. A CT scan revealed gastric pneumatosis and portal venous air, prompting urgent diagnostic laparoscopy and intraoperative esophagogastroduodenoscopy, which showed patchy mucosal ischemia without transmural involvement. Blood cultures identified *Pseudomonas* and *Klebsiella oxytoca*, which were treated with cefepime and metronidazole, alongside oral vancomycin for *Clostridioides difficile*. The patient recovered without EG-related complications over four weeks and was discharged to home hospice. Emphysematous gastritis is not commonly reported in the setting of chemotherapy for colon cancer. This case highlights the efficacy of conservative management in EG and underscores the need for heightened awareness of this condition in oncologic patients receiving chemotherapy.

## Introduction

Emphysematous gastritis (EG) is an uncommon pathology identified radiologically as air in the stomach wall secondary to gas-producing bacteria, often accompanied by portal venous air. Gram-negative, gram-positive, fungal, and anaerobic organisms have all been reported in biopsies of stomachs with this finding, with the most common causative organisms being *Escherichia coli*, *Streptococcus* species, *Enterobacter* species, *Pseudomonas aeruginosa*, *Clostridium* species, *Klebsiella pneumoniae*, *Staphylococcus aureus*, and *Candida* species [1]. Emphysematous gastritis is life-threatening, with early identification and treatment being key to preventing clinical deterioration. The mortality rate has been described as 55% by Matsushima et al. and 60% by Al-Jundi et al., with most successful treatments being non-surgical [2,3]. This case report describes a patient treated successfully with conservative management, including photographic documentation from an early diagnostic laparoscopy and intraoperative esophagogastroduodenoscopy (EGD).

## Case presentation

**Figure 1:**
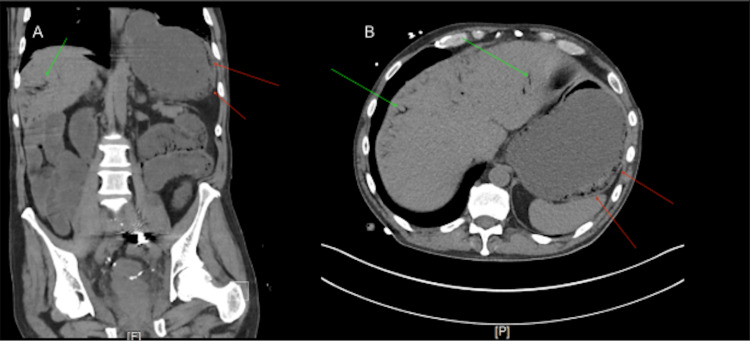
The CT scan demonstrating the coronal (A) and axial (B) views. Red arrows: Air within the stomach wall secondary to gas-producing bacteria. Green arrows: Portal venous gas translocation of air from the stomach wall into portal hepatic circulation.

A 50-year-old male with a past medical history of stage 4 colon cancer treated with sigmoidectomy and radiation, ongoing chemotherapy with cytarabine, a colovesicular fistula with bilateral nephrostomy tubes, recurrent small bowel obstruction (SBO), and a recent colostomy parastomal hernia repair presented to the hospital after being found on the floor by his family with altered mental status. The patient met sepsis criteria, exhibiting hypotension with a systolic blood pressure of 84 mmHg and a heart rate of 102 bpm, along with a lactic acid level of 6.2 (0.4-2.0 m/mol) and a white blood cell count of 18.7 × 10^9/L (4.5-10.9 k/uL). On examination, the patient was soft, distended, and non-tender, with melena in his ostomy bag. Empiric broad-spectrum antibiotics were initiated to cover the most likely gas-forming organisms, and the patient received 5.5 liters of fluid over the first 12 hours. An initial CT scan of the abdomen demonstrated gastric pneumatosis with accompanying portal venous air, prompting urgent surgical consultation (Figure 1).

**Figure 2:**
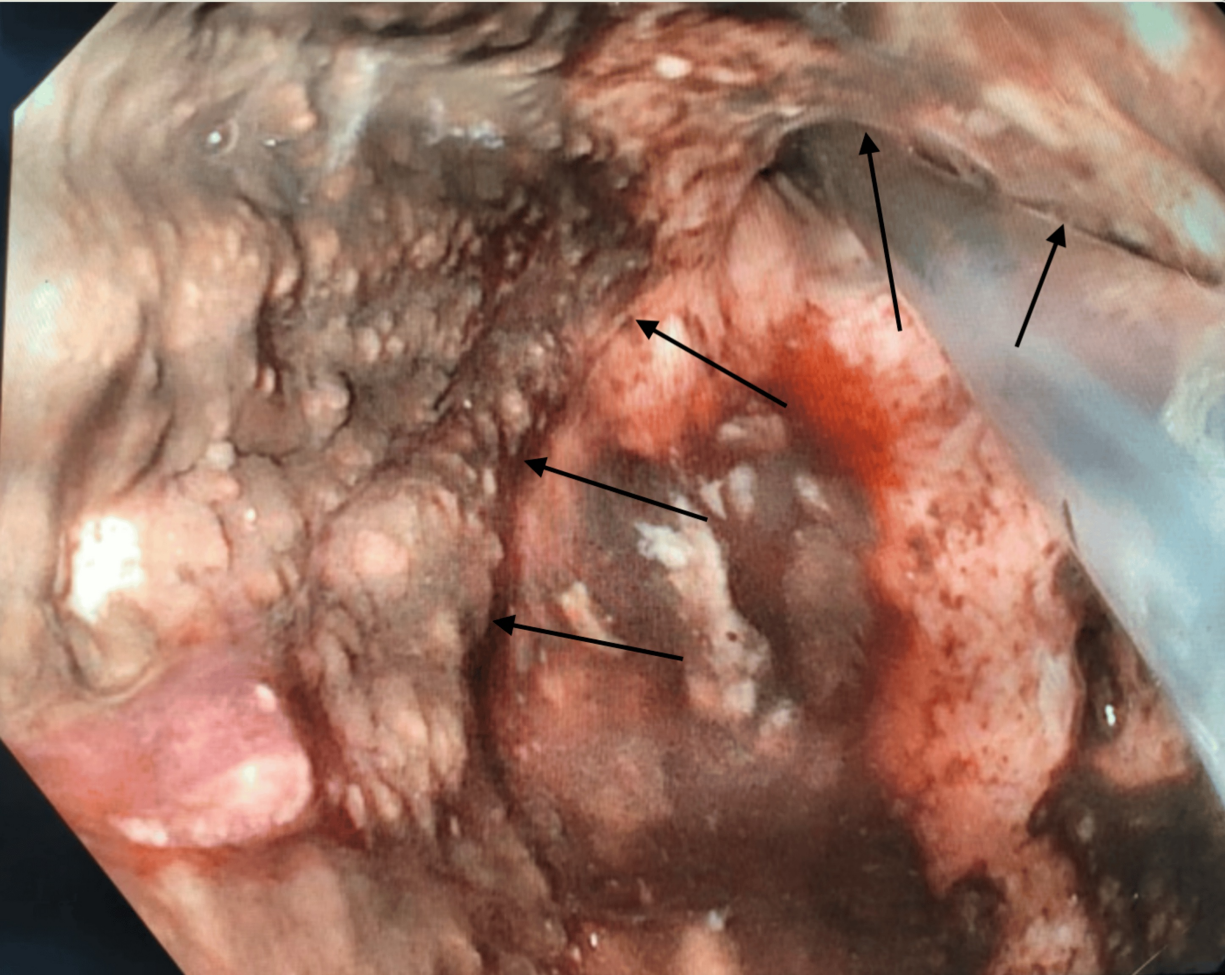
Intraoperative EGD revealing patchy ischemic necrosis of mucosa of the stomach in the retroflexed position (black arrows). EGD: Esophagogastroduodenoscopy

Due to concern for acute gastric ischemia, the patient was taken urgently to the operating room for diagnostic laparoscopy, which revealed no acute findings. An intraoperative EGD was performed, showing patchy mucosal ischemia of the stomach and lower esophagus (Figure 2). With no signs of transmural ischemia, the stomach mucosa was biopsied, and the patient was returned to the surgical ICU for recovery. Early in his hospital course, blood cultures resulted positive for Pseudomonas and Klebsiella oxytoca, which were treated by narrowing his antibiotic regimen to cefepime and metronidazole. He was also found to be positive for Clostridioides difficile, which was treated with oral vancomycin. The patient recovered well over a period of 4 weeks without complications related to his EG. At the end of his hospital stay, he was made do-not-intubate (DNI)/do-not-resuscitate (DNR) and discharged to home hospice.

## Discussion

In the literature, EG has historically been a rare pathology, with only 57 case reports published between 1980 and 2018 [3]. However, more recently, many additional publications on the topic have appeared, including 40 case reports between 2019 and 2022 [4]. Chemotherapy has also been established as a risk factor for EG. However, through our literature review, we have found only one other case of EG in the setting of active chemotherapy for colon cancer (Table 1) [5,6]. While cytarabine chemotherapy is implicated as the primary predisposing factor, this patient's recent abdominal surgery and history of recurrent SBO may have contributed to compromised mucosal integrity or bacterial overgrowth, creating a multifactorial pathway to EG.

**Table 1:** Comparison of cited EG cases EG: Emphysematous gastritis

Author	Malignancy	Chemotherapy agents	Causative organism	Management	Outcome
Johnson et al. (the current report)	Colon adenocarcinoma stage 4	Cytarabine	*Pseudomonas* and *K. oxytoca*	Conservaitve	Survived
Ogbue et al. [5]	Colon adenocarcinoma stage 4	Leucovorin, fluorouracil, irinotecan, and bevacizumab	Not provided	Conservaitve	Survived
Savvala et al. [6]	Breast Cancer	Paclitaxel	Clostridium ventriculi	Total gastrectomy	Survived

Chemotherapy can contribute to the development of EG through mechanisms such as immunosuppression and direct damage to the gastric mucosal barrier, thereby facilitating invasion by gas-producing bacteria. Agents like cytarabine, which the patient in this case was receiving, are known to cause neutropenia and gastrointestinal toxicity, potentially exacerbating vulnerability in already immunocompromised individuals with advanced malignancies. For instance, bevacizumab has been implicated in a case of EG in metastatic colon cancer, possibly due to its anti-angiogenic properties leading to mucosal ischemia and impaired healing [5]. Similarly, paclitaxel therapy for breast cancer has been associated with EG induced by *C. ventriculi,* where chemotherapy-induced mucosal injury allowed bacterial proliferation, necessitating surgical intervention such as total gastrectomy [6]. Additionally, transarterial chemoembolization (TACE) for hepatocellular carcinoma has been linked to EG, likely through embolic effects or localized toxicity affecting gastric integrity, though conservative management proved successful in one reported instance [7]. These examples illustrate that while EG remains rare, its association with chemotherapy is increasingly documented, emphasizing the need for heightened awareness in oncologic patients undergoing such treatments, particularly when combined with radiation or other predisposing factors.

Emphysematous gastritis is diagnosed by observing gas in the stomach wall, but the classification can be confusing due to overlapping diagnoses. Gas within the stomach wall is a rare occurrence. When air or gas is present on imaging, gastric emphysema, EG, or pneumatosis intestinalis may be diagnosed. The presence of gas must originate from the environment or be produced within the gastric wall [8].

Emphysematous gastritis is a form of phlegmonous gastritis in which the bacteria embedded in the stomach wall produce gas. These microorganisms penetrate the local mucosa or spread through hematogenous dissemination from another source, causing this condition. The stomach is an uncommon site of involvement due to its abundant blood supply, gastric acidity, and mucosal barrier [9]. Emphysematous gastritis occurs when these defenses are compromised, such as in peptic ulcer disease, alcohol abuse, diabetes, or immunosuppression. Other associated factors include non-steroidal anti-inflammatory drug use, gastroenteritis, and recent gastric surgery [10].

Patients typically present with fever, acute abdominal pain, shock, and often hematemesis. A late presentation may include vomiting necrotic casts of stomach mucosa, which is considered a pathognomonic sign. The gas-forming organisms infiltrate the stomach wall diffusely. The necrotic cast results from dissection along the plane of the muscularis mucosa by invading organisms, leading to mucosal erosions, acute inflammation, edema, and intramural gas. Over time, the inflammation of the wall of the stomach causes granulation tissue formation by fibroblasts [10-13].

Characteristic radiographic findings for EG include a thickened wall with intramural gas and a dilated stomach. The gas from the wall of the stomach is also often seen in the portal venous system within two days of symptom onset and can be present for months after symptom resolution. The gas in the stomach wall may sometimes be appreciated on abdominal x-ray, but the best method for detecting a smaller amount of intramural air is CT, which also allows for localization of the gas as well [10,14,15].

Early recognition and treatment of EG play a crucial role in survival. This condition carries a mortality rate of 55% to 61%. Acute-phase treatments include hemodynamic stabilization with resuscitative intravenous fluids, empiric antibiotics covering anaerobes and gram-negative organisms, bowel rest, and identification and resolution of predisposing factors. Current recommendations advise avoiding surgery in the acute phase unless perforation is present, but optimal therapy has not been definitively established. The existing literature does not support gastric resection during the acute phase of EG as standard therapy [3,10,13,16].

## Conclusions

This case report highlights the successful conservative management of EG in a 50-year-old patient with stage 4 colon cancer undergoing cytarabine chemotherapy. Despite the high mortality rate associated with EG, prompt diagnosis through imaging, targeted antibiotic therapy, and supportive care led to the patient’s recovery without complications related to EG. The rarity of EG in the context of chemotherapy, particularly in colon cancer, suggests a potential link warranting further investigation. This case underscores the importance of early recognition and non-surgical treatment strategies in managing EG, especially in immunocompromised oncologic patients.
